# rLOAD: does sex mediate the effect of acute antiplatelet loading on stroke outcome

**DOI:** 10.1186/2042-6410-5-9

**Published:** 2014-07-15

**Authors:** Dawn M Meyer, Jo-Ann Eastwood, M Peggy Compton, Karen Gylys, Justin A Zivin

**Affiliations:** 1 UC San Diego School of Medicine, 200 W Arbor Drive, MON, Suite 3, San Diego, CA 92103-8466, USA; 2 UCLA School of Nursing, 700 Tiverton Ave, Los Angeles, CA, 90095, USA; 3 Georgetown University School of Nursing and Health Studies, Washington, DC 20007, USA; 4 UC San Diego School of Medicine, 200 W Arbor Drive, MON, Suite 3, San Diego, CA 92103-8466, USA

**Keywords:** Acute stroke, Stroke model, Antiplatelet, Aspirin

## Abstract

**Background:**

Biologic sex can influence response to pharmacologic therapy. The purpose of this proof-of-concept study was to evaluate the medicating effects of estrogen in the efficacy of acute antiplatelet loading therapy on stroke outcome in the rabbit small clot embolic model.

**Methods:**

Female and male (20/group) New Zealand White rabbits were embolized to produce embolic stroke by injecting small blood clots into the middle cerebral artery via an internal carotid artery catheter. Two hours after embolization, rabbits were treated with standard dose antiplatelet loading (aspirin 10 mg/kg plus clopidogrel 10 mg/kg). Primary outcome measures were platelet inhibition, behavioral outcome *P*_50_ (the weight of microclots (mg) that produces neurologic dysfunction in 50% of a group of animals), and effect of endogenous estrogen on outcome.

**Results:**

For the first time in a non-rodent model of stroke, it was found that higher endogenous estrogen levels resulted in significantly better behavioral outcome in female subjects (*r*_s_ −0.70, *p* < 0.011). Platelet inhibition in response to collagen, arachidonic acid, and adenosine diphosphate (ADP) was not significantly different in females with higher vs. lower estrogen levels.

**Conclusions:**

Behavioral outcomes are improved with females with higher endogenous estrogen levels treated with standard dose antiplatelet loading. This is the first non-rodent study to demonstrate that higher endogenous estrogen levels in female rabbits appear to be neuroprotective in ischemic stroke. This research supports the further study of the effect of endogenous estrogen levels on outcome with standard dose antiplatelet loading in stroke patients not eligible for revascularization therapies.

## Background

Women experience 55,000 more strokes annually than men, are 30% less likely to receive recombinant tissue plasminogen activator (rt-PA), and have poorer outcomes when not acutely treated [1-4]. In addition, biologic sex may mediate response to pharmacotherapies [5]. Molecular, animal, and clinical studies have clearly demonstrated that biologic sex and endogenous sex steroids influence stroke outcome [6]. Estrogen appears to provide neuroprotection, with molecular, histological, and behavioral outcomes being superior in infarcted pre-menopausal females compared to both age-matched male and post-menopausal or ovarectomized female subjects [7,8]. The role of endogenous estrogen and sex-specific responses must be considered when developing new treatment strategies to ensure that women receive efficacious treatment.

The purpose of this study was to evaluate the efficacy of antiplatelet loading and the effects of endogenous estrogen on stroke outcome in female subjects in the rabbit small clot embolic model (RSCEM). Previous dose escalating work in exclusively male rabbits in this model found that acute antiplatelet loading with aspirin 10 mg/kg plus 10 mg/kg clopidogrel had the most significant benefit on stroke outcome in the RSCEM [9]. Using the male group from the previous work, this examined the mediating effects of endogenous estrogen on stroke outcome with standard dose antiplatelet loading (aspirin 10 mg/kg plus 10 mg/kg clopidogrel) on the primary outcomes of (1) inhibition of platelet aggregation and (2) the relationship between endogenous serum estrogen and behavior outcome in female animals.

## Methods

### Design and sample

This was a blinded study of the mediating effects of endogenous estrogen in acute antiplatelet loading in the RSCEM. Animals (20 females, 2–4 kg, 1 year old, New Zealand White rabbits) were purchased from Rabbit Source, Ramona, CA, USA. Rabbits were supplied food (alfalfa cubes) and water *ad libitum* while under quarantine in an enriched environment for at least 3 days before experimental use. The University of California Los Angeles and Veterans Administration San Diego Health System (VASDHS) Institutional Animal Care and Use Committees (IACUC) approved the surgical and treatment procedures used in this study (Modification #5 Protocol #07-043).

### Procedures

All surgical, embolization, and histological procedures were done based on the techniques of Zivin and Lapchak [10-12]. The primary investigator unblinded to sex was responsible for administering the stroke and treatment; trained laboratory technicians blinded to sex and treatment assessed the primary outcomes. Care was used throughout the study to minimize pain and discomfort. Per the IACUC approved protocol, rabbits were euthanized if they showed extreme discomfort or were unable to reach food or water.

#### Surgical procedures

Surgery was done in a controlled environment (22.8°C–23.2°C), and all procedures were based on the techniques of Zivin and Lapchak [10-12]. The RSCEM has been utilized successfully in stroke research as it has been used to test pharmacologic interventions such as recombinant tissue plasminogen activator (rt-PA), NXY-059, tenecteplase (TNK), and microplasmin [13-16]. The rabbit remained conscious during clot administration and treatment to more accurately mimic the human ischemic stroke condition. In addition, a validated clinical rating score for RSCEM outcomes has been developed, allowing researchers to quantify neurologic behavioral responses in the model (Table 1) which has been shown to have a <5% inter-rater variability providing a consistent and easy measurement of clinical outcome [11].

**Table 1:** The RSCEM clinical rating scale

	**Observation**
Reaction to embolization	__Mild (nystagmus, withdrawal)
	__Moderate (kicking)
	__Severe (rolling, vocalization)
	__Death
RSCEM rating	__Normal (score = 0)
	__Abnormal (score = 1)
	Place a check next to the abnormal behavior exhibited:
	__Ataxia
	__Leaning
	__Circling
	__Lethargy
	__Nystagmus
	__Loss of balance
	__Loss of limb or facial sensation
	__Paraplegia
	__Death

A catheter is surgically implanted for injection of the small clot. Rabbits were anesthetized with isoflourane (5% induction, 3% maintenance) by face mask, the bifurcation of the right carotid artery was exposed, and the external carotid was ligated just distal to the bifurcation. A catheter was inserted into the common carotid artery and advanced to the internal carotid artery, secured with ligatures, and the distal end left accessible outside the neck. The rabbits were allowed to recover from anesthesia for a minimum of 2 h until they were awake and behaving normally as evidenced by no identified persistent behaviors from the RSCEM behavior scale prior to embolization (Table 1).

#### Preparation and administration of small clot embolism

Blood drawn from a donor rabbit was allowed to clot at 37°C and suspended in Dulbecco's phosphate buffered saline (PBS) solution containing 0.1% bovine serum albumin and emulsified with a Polytron small particle cutter (Kinematica GmbH, Kriens-Luzern, Sweden). Clots were sized by sequential filtration through a 240-μm^2^ screen and a 105-μm^2^ nylon net; those retained were washed with PBS and allowed to settle. This supernatant was then removed and the clot particles labeled with tracer quantities of 15-μm radiolabeled microspheres (cobalt-57) to enable quantification of clot weight after sacrifice. PBS solution was then added to the clot particles so that clot particles were suspended in 1 ml, which was drawn into a syringe for administration.

Clot particles were rapidly administered through the intra-arterial injection catheter, and the system was flushed with normal saline ensuring that no air bubbles were present that may cause air embolism. Animals that died or were euthanized after embolization and treatment were included in the study.

#### Drug administration

Animals were treated with a standard loading dose of antiplatelets (aspirin 10 mg/kg plus 10 mg/kg clopidogrel) at 2 h post-embolization. Time of administration was consistent with the time at which rt-PA administration is no longer feasible [17]. Medication was administered as an intravenous (IV) injection into an ear vein with reconstituted acetylsalicylic acid (ASA) plus clopidogrel no sooner than 5 min after reconstitution. Powdered drug was obtained from Sigma-Aldrich (St. Louis, MO, USA) and reconstituted in vehicle (Cavitron, Fisher Scientific, Pittsburgh, PA, USA) per manufacturer's instructions and diluted to a cubic centimeter volume. The per kilogram doses of drugs reflect standard antiplatelet doses in clinical practice [ASA 325 mg (10 mg/kg); clopidogrel 300 mg (10 mg/kg)] [18,19].

### Outcome measures

#### Platelet aggregation

Blood was collected at pre-stroke baseline, 3 h, 6 h, and 24 h by puncture of an ear vein into a 3.8% trisodium citrate solution (1–2 cm^3^). Platelet aggregation was tested via optical, whole blood single-channel Chrono-log aggregometer [20]. Responses to adenosine diphosphate (ADP), arachidonic acid (AA), and collagen-induced aggregations (concentrations, 2.5 μM, 250 μM, and 12 μg/ml, respectively) were assessed. Platelet aggregation was recorded in regard to the change of impedance (Ω) and was reported as the percent decrease from baseline aggregation. The platelet aggregation measurements in whole blood samples will be examined by an impedance aggregometer (Model 590, Chrono-log Corporation, Havertown, PA, USA) using the AggroLink software package. The system detects the change of electrical impedance due to the adhesion and aggregation of platelets on the electrode's surface in the test cuvette. Impedance aggregometry testing was performed between 60 and 180 min after drawing blood. The measurements were carried out at 37°C. Citrate blood was diluted 1:1 with 0.9% sodium chloride and pre-warmed. Aggregation to collagen, AA, and ADP was monitored for 6 min. The results are reproducible with a variability of <10%.

#### Endogenous estrogen

Sex was operationalized by circulating levels of estrogen (17β-estradiol) in the female rabbits. Levels of 17β-estradiol were collected immediately prior to embolization, and analyses were performed by the University of California San Diego Research Laboratory Services using the valid and reliable Sorin kit (sensitivity = 5 pg/ml, s.e. 0.006 pg/ml, *r* = 0.90 to direct assay) [21].

### Power analysis

All power analyses were done using PASS 2008 version 08.0.6. Sample sizes were selected to provide 80% power at a two-tailed significance level of 0.05 in two groups. For platelet aggregation, assuming a large effect size of estrogen based on the Loading of Aspirin and Clopidogrel (LOAD) study (*d* = 0.80) [22], a sample size of 20 was required to detect a 20% difference between the groups.

### Statistical analysis

All statistical analyses were done using SPSS version 15.0 or later. A *p* value of <0.05 was considered significant. Platelet aggregation inhibition was evaluated via a *t* test analysis. A Spearman rank correlation was used to examine the relationship of estrogen level and outcome in female subjects. Variances are reported in standard deviation*.*

## Results

There were no statistically significant differences among the subjects with respect to age, weight, surgical time, or body temperature during surgery. 17β-estradiol levels in the females ranged from 36.9 to 61.1 pg/ml (mean 44.1 ± 6.0 pg/ml). One subject died post-embolization but before treatment and was replaced in the sample.

### Inhibition of platelet aggregation

At all timepoints, platelet aggregation in response to collagen, AA and ADP were not significantly different in females based on endogenous estrogen level (*r*_s_ −0.31, *p* = 0.26)*.* There were no significant differences in platelet aggregation between historical male and current female subjects (Table 2)*.*

**Table 2:** Stroke outcome measures by sex

**Inhibition of platelet aggregation**	**Male (historical comparison)**	**Female**	***p*** **value**
3 h			
Collagen	33.2% (±12.2)	34.6% (±10.6)	0.53
AA	19.5% (±8.5)	22.7% (±15.1)	0.09
ADP	38.5% (±4.7)	37.5% (±6.6)	0.69
6 h			
Collagen	65.1% (±10.1)	65.6% (±8.6)	0.78
AA	43.0% (±9.2)	42.7% (±7.7)	0.86
ADP	64.9% (±5.4)	66.8% (±7.4)	0.27
24 h			
Collagen	71.1% (±11.6)	70.0% (±14.6)	0.51
AA	58.8% (±8.7)	56.6% (±5.8)	0.09
ADP	76.0% (±16.3)	74.4% (±9.6)	0.25

Inhibition of platelet aggregation was measured as change of impedance (Ω) in percentage from baseline platelet aggregation levels. Aggregation was measured in reaction to collagen, arachidonic acid (AA), and adenosine diphosphate (ADP).

### Estrogen

**Figure 1:**
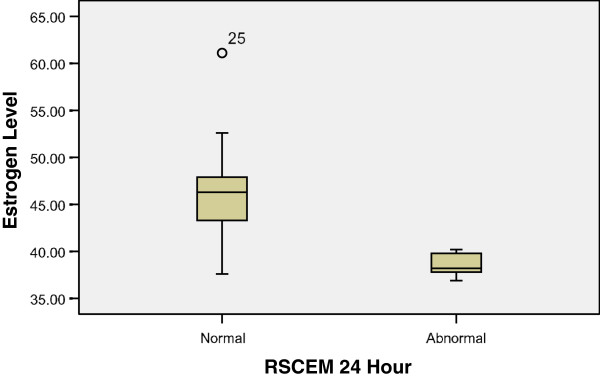
**Estrogen and behavioral outcome.** Females with an RSCEM score of 0 (normal) had significantly higher estrogen levels as compared to females with a score of 1 (abnormal score) at 24 h (46.5 pg/ml vs. 38.5 pg/ml, 95% CI 3.2–12.9, *p* = 0.003). Higher estrogen levels were significantly associated with higher *P*_50_ at 24 h (*r*_s_ −0.70, *p* < 0.011).

Although serum levels of endogenous estrogen in female animals were not significantly associated with inhibition of platelet aggregation at any timepoint or reagent, they were significantly and positively associated with behavioral outcome. Females with an RSCEM score of 0 (normal) had higher baseline endogenous estrogen levels as compared to females with a score of 1 (abnormal score) (46.5 pg/ml vs. 38.5 pg/ml, 95% CI 3.2–12.9, *p* = 0.003). Higher estrogen levels were associated with higher *P*_50_ at 24 h (*r*_s_ −0.70, *p* < 0.011) (Figure 1).

## Discussion

Antiplatelet medications have been utilized in cardiac and cerebrovascular occlusive diseases for primary prevention of thrombotic events and secondary prevention of future events [23-25]. The acute use of these medications concomitantly in large loading doses to acutely treat stroke has only recently garnered empirical attention [22,26]. The purpose of this study was to assess for the mediating effects of endogenous estrogen in female subjects on stroke outcome after acute antiplatelet loading in the RSCEM. In this pilot study of standard dose antiplatelet loading after stroke, there was a significant, positive association between endogenous estrogen levels and neurologic outcomes in female rabbits. These neuroprotective effects appear to be hormone, rather than biologic sex, mediated and support effects seen in other species.

Estrogen has been shown to have multiple neuroprotective effects in the literature. Neuroprotective effects include (1) preservation of regional blood flow via amplification of nitric oxide signaling and activation, (2) reduction of intravascular leukocyte adhesion, and (3) anti-apoptotic activity via caspase-mediated cell death [27]. Estrogen preserves regional blood flow via endothelial vasodilatation due in part to nitric oxide and prostanoid release, resulting in cerebrovascular dilatation, increased cerebral blood flow, and decreased ischemic injury [28]. Estrogen has also been shown to decrease leukocyte adhesion and inhibit the expression of adhesion molecules, thus minimizing the pro-inflammatory damage [29,30]. Further studies of antiplatelet medications in female models of stroke are warranted to assess these processes.

Beyond neuroprotection, the correlation of estrogen with a normal neurologic outcome seen in this study may be related to its anti-apoptotic properties and its vascular effects. Recent data suggest divergent pathways of ischemic cell death in females vs. males. Under conditions of cerebral hypoxia, the neuronal tissue of males is more susceptible to poly(ADP-ribose) polymerase (PARP)-mediated apoptosis than that of females [31]. Estrogen also increases mitochondrial efficiency in the ischemic brain, providing protection from caspase-mediated cell death via the reduction of free radical production while stimulating angiogenesis [32-34]. The most significant vascular effect of estrogen, with respect to stroke, may be its ability to cause vasodilatation in the endothelium and increase blood flow [35]. The ability to increase perfusion in the cerebral arteries may provide tissue-sustaining perfusion to the brain tissue at risk for hypoxic death. Though beyond the scope of this study, it cannot be ruled out that standard antiplatelet loading potentiates the anti-apoptotic and/or vascular effects of estrogen. While it is not hypothesized that estrogen mediates or modifies the mechanism of action of either clopidogrel or aspirin, the authors propose that there is a synergistic effect of inhibiting platelet activity and providing neuroprotection by both the medications and endogenous estrogen leading to a decrease in ischemic damage. The small sample size of females with higher 17-β-estradiol levels did not allow for a statistically sound comparison between the females with high estrogen levels and males in this study. Future studies will include larger samples to assess this effect.

While the administration of exogenous estrogen has been extensively tested for its effect on stroke development and outcome [36-40], limited work has been done examining the role of endogenous estrogen in stroke pathology and outcomes. Moreover, little clinical work has addressed endogenous estrogen levels in stroke risk or outcome [41,42]*.* This study aimed to correlate endogenous estrogen levels and behavioral outcome in post-pubescent, pre-menopausal female rabbits. Animal (primarily rodent) and natural history studies show that endogenous estrogen is protective in cerebral ischemia [43,44]. Endogenous estrogen levels have been positively correlated with increases in beneficial antioxidant enzymes, decrease in lactate dehydrogenase (LDH) activities, and decrease in leukocyte adhesion in the ipsilateral hemisphere [40]. Future antiplatelet loading studies in this model must examine these markers of improved stroke outcome.

This study found that endogenous estrogen levels were positively correlated with a normal neurologic outcome, supporting the current literature's finding that higher endogenous estrogen levels lead to smaller infarct volumes [38]. Providing strong evidence for the role of endogenous estrogen, several other studies have shown that the neuroprotective effects of estrogen, described above, can be abolished by ovariectomy [45-48] or by declining estrogen levels during reproductive quiescence resulting in effects that mimic male outcomes [49]. Further testing must be done to compare stroke outcome in pre-pubescent and post-menopausal rabbits in the mediation of response to antiplatelet loading.

Limitations to this study exist. Estrogen level was not manipulated in this study and showed only moderate variance in the sample. This study was powered to reflect a large effect size for the variable of sex. It is likely that the effect of sex may be either small or medium and may require a larger sample size to elicit. In addition, the lack of variance within this sample may have made it more difficult to detect a significant difference in outcome related to either very low or very high endogenous levels. Subjects with ovariectomy or receiving estrogen treatment must be studied to fully characterize its effect on outcome. Another limitation was the use of young, healthy rabbits. Future studies must be designed to further explore the efficacy of acute antiplatelet therapy and the role of endogenous estrogen and other sex steroids in older animals with typical stroke co-morbidities. This study did not employ a control group or dose-escalation studies in the female subjects. Because this was a pilot study, we were interested in comparing the ‘best dose’ of antiplatelet therapy in the female group as a first step. Future studies will include a full comparison of each group.

Despite compelling evidence for a neuroprotective role of estrogen in cerebral ischemia, complete examination of the relationship requires consideration of non-estrogen-related physiological sex differences. Prospective studies of the role of exogenous and endogenous progesterone, the androgens, and chromosomal differences are still required.

## Conclusions

This is the first study to examine the mediating effects of endogenous estrogen on stroke outcome in acute antiplatelet loading treatment in a non-rodent species. Trials focusing on sex and hormonal differences with all stroke therapies must be explored to assess for differential benefits in men vs. women. It is vital that the role of endogenous estrogen be explored with respect to novel stroke therapies. Only this method of assessment will allow researchers to glean an accurate assessment of the independent effect of sex-related variables and the interactive effects of these variables with stroke treatment. This research supports the further study of the effect of endogenous estrogen levels on outcome with standard dose antiplatelet loading in stroke patients not eligible for revascularization therapies.

## Competing interests

The authors declare that they have no competing interests.

## Authors' contributions

DM conceived the concept, designed the study, performed the experiments, collected and analyzed data, and prepared the manuscript. JE and KG refined the study design, prepared the manuscript, and critically edited the manuscript. PC and JZ participated in the study design, prepared the manuscript, monitored data collected, analyzed the data, and critically edited the manuscript. All authors read and approved the final manuscript.
